# Patients With Atypical Chest Pain: Epidemiology and Reported Consequences

**DOI:** 10.7759/cureus.53076

**Published:** 2024-01-27

**Authors:** Mandreker Bahall, Sherece Kissoon, Samiha Islam, Sarah Panchoo, Naomi Bhola-Singh, Mitra Maharaj, Fiyad Khan, Sathyadeep Marajh, Aishwarya Maharaj, George Legall

**Affiliations:** 1 Caribbean Center for Health Systems Research and Development, University of the West Indies, St. Augustine Campus, Marabella, TTO; 2 Faculty of Medical Sciences, University of the West Indies, Eric Williams Medical Sciences Complex, San Juan, TTO

**Keywords:** loneliness, depression, stress, anxiety, atypical chest pain

## Abstract

Background: Approximately a quarter of the global population experiences chest pain during their lifetime worldwide. Although largely non-life-threatening, many patients experience mental, physical, social, and financial consequences.

Aim: This study aimed to describe and determine the epidemiology and consequences of patients presenting with atypical chest pain (ACP).

Method: Data were obtained from 102 participants, from a desired sample size of 166. The target population was patients who presented with ACP at the Accident and Emergency Department of a Teaching Hospital in Trinidad during a two-year period, from January 2021 to December 2022. The data collection instrument used was a 34-item online questionnaire. Data were analyzed using both descriptive and inferential statistical methods.

Results: Participants were predominantly women (63.7%; n = 65), between 31 and 50 years of age (74.5%; n = 76), in full-time employment (n = 58; 56.9%), who lived with at least one person (90.2%; n = 92) at the time of the episode. Overall, 61.8% (n = 63) reported having a stressful life. Hypertension (30.4%; n = 31) and diabetes (18.6%; n = 19) were the leading comorbidities. Participants experienced mild to severe anxiety (53.9%; n = 55), moderate to severe depression (25.5%; n = 26), moderate stress (65.7%; n = 67), and loneliness (25.5%; n = 26). A stressful life was associated with, and was a predictor of, both anxiety and loneliness. No sociodemographic variables were associated with depression or stress. The most common self-reported consequences were "fear as a result of the pain" (68.6%; n = 69), "interruptions to daily life" (60.8%; n = 61), "reduction in time spent on hobbies" (62.7%; n = 63), and costly diagnostic/investigative tests (62.7%; n = 64). The majority of patients (52.9%; n = 53) reported reduced quality of life. The most common treatment prescribed was paracetamol (53.9%; n = 55) and exercise (23.5%; n = 24).

Conclusion: The study participants were mainly women, 31-50 years old, who had experienced anxiety, stress, or depression. They mainly experienced fear and self-reported a reduced quality of life.

## Introduction

Non-cardiac chest pain (CP) is referred to as atypical chest pain (ACP) or chest pain of unknown cause. In the US emergency department (ED), “from 2006 to 2016, there were 42.48 million chest pain visits” [1]. ACP accounts for almost 49-60% of emergency medical admissions presenting with acute central chest pain [2]. Spalding et al. reported that 20-30% of all emergency medical admissions were attributable to acute central chest pain, with 49-60% of those categorized as atypical [2]. Capewell and McMurray [3] found that more than 50% of patients presenting to the clinic with chest pain (CP) had a low risk for coronary heart disease. Due to uncertainty, the diagnosis is often recorded as atypical chest pain, non-cardiac chest pain, or “chest pain of unknown cause” [4], with its etiology attributed to “anxiety” or “panic.” Despite the prevalence of ACP, Eslick [5] lamented the lack of clarity regarding the impact and natural history of non-cardiac chest pain in the community. This has tended to result in suboptimal management as well as compromised quality of life and overall productivity. This study aimed to identify the factors associated with and predictors of ACP and the consequences of ACP. 

## Materials and methods

**Figure 1:**
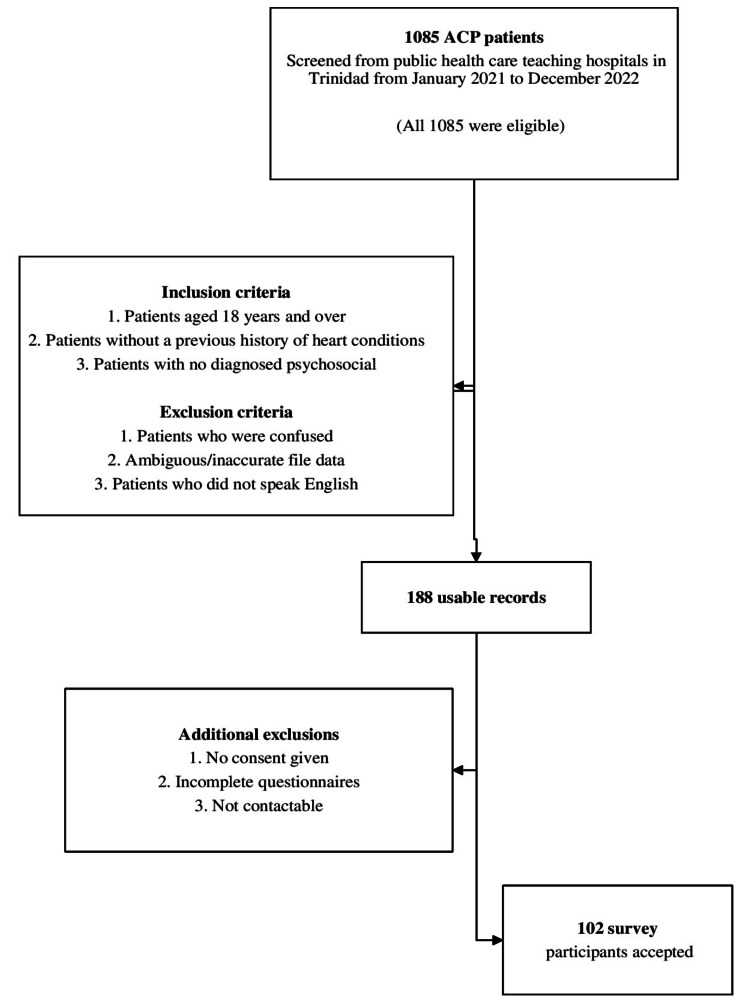
Sample selection and participants. ACP: atypical chest pain.

This study was a cross-sectional cohort of patients presenting with atypical chest pain (ACP) at the Accident and Emergency (A&E) Department of a Teaching Hospital in Trinidad and Tobago. The inclusion criteria were as follows: (i) at least 18 years of age, and (ii) a resident or citizen of Trinidad and Tobago. The exclusion criterion was difficulty communicating, not contactable, and missing data. Records from the A&E Departments of the two hospitals showed that, during the study period, 1085 patients presented with the criteria for non-cardiac chest pain. Of these, 17.3% (n = 188) were usable (could be located, no key missing data, no restricted access), and 9.4% (n = 102) of the target population were accepted for analysis (Figure 1).

The data collection instrument was a pre-tested 34-item self-completed questionnaire comprising demographic data (nine questions); medical history (two questions); atypical chest pain factors (11 questions), psychological instruments (four questions) from the General Anxiety Disorder-7 (GAD-7) [6], University of California, Los Angeles 3-Item Loneliness Scale (UCLA-3) [7], Patient Health Questionnaire-9 (PHQ-9 (Depression)) [8], and Perceived Stress Scale (PSS-10) [9]; consequences of atypical chest pain (five questions); and investigations (three questions). Data were collected from 8 May 2023 to 23 August 2023 inclusive, via telephone and analyzed using SPSS Version 23 (IBM Corp., Armonk, NY, USA) and MINITAB Version 21 (Minitab, State College, PA, USA). Cronbach alpha was used to assess the reliability (internal consistency) of each of the four psychological instruments (anxiety, depression, loneliness, and stress). Data analysis included descriptive and inferential statistical methods. The former included frequency and percentage distribution tables and means and standard deviations. Inferential methods included 95% confidence intervals (CI), hypothesis testing (Chi-square tests of association, analysis of variance (ANOVA), Tukey’s pairwise multiple comparisons), and logistic regression (ordinal and binary). All hypotheses were tested at the 5% level of significance.

Ethical approval was obtained from the Ethics Committee of the University of the West Indies (UWI) and the Regional Health Authority (RHA), which had jurisdiction over each of the three teaching hospitals. Verbal consent to participate was obtained from all participants.

## Results

Characteristics of patients with ACP

Patient Profiles

**Figure 2:**
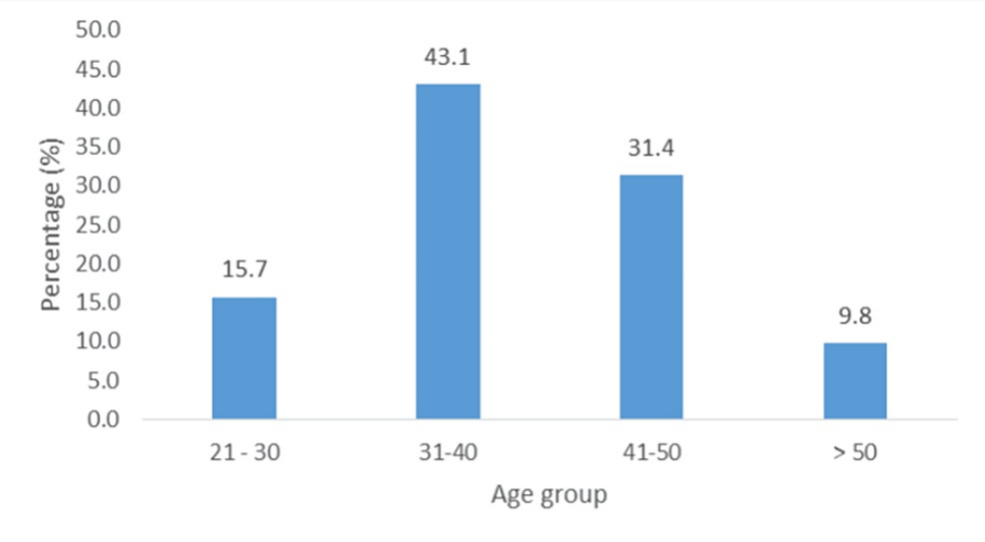
Participant age group distribution.

**Figure 3:**
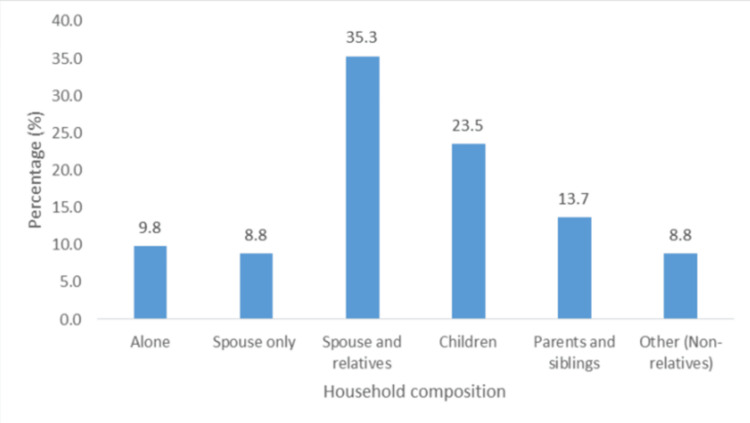
Household composition of participants.

About 63.7% (n = 65) of participants were mainly women, 12.7% (n = 13) did not have more than a primary school education, 29.4% (n = 30) were unemployed, and 36.2% (n = 37) earned less than TT $5000 (Table 1). The majority were middle-aged, between 31 and 50 years old (74.5%; n = 76) (Figure 2). Only 9.8% (n = 10) of participants lived alone, and 35.3% (n = 36) lived with a spouse or other relatives (Figure 3), while 90.2% (n = 92) lived with at least one person at the time of the episode. In addition, 18.6% (n = 19) had social support, and 33.3% (n = 34) had financial support.

**Table 1:** Frequency and percentage distribution.

Variable	n	%
Sex		
Male	37	36.3
Female	65	63.7
Education		
Primary	13	12.7
Secondary	46	45.1
Tertiary	43	42.2
Monthly income (TTD)		
None	23	22.6
<2500	13	12.7
2500-5000	24	23.5
5000-7500	15	14.7
7500-10,000	20	19.6
>10,000	7	6.9
Employment status		
Unemployed	30	29.4
Part-time	14	13.7
Full time	58	56.9

Lifestyle Habits

**Figure 4:**
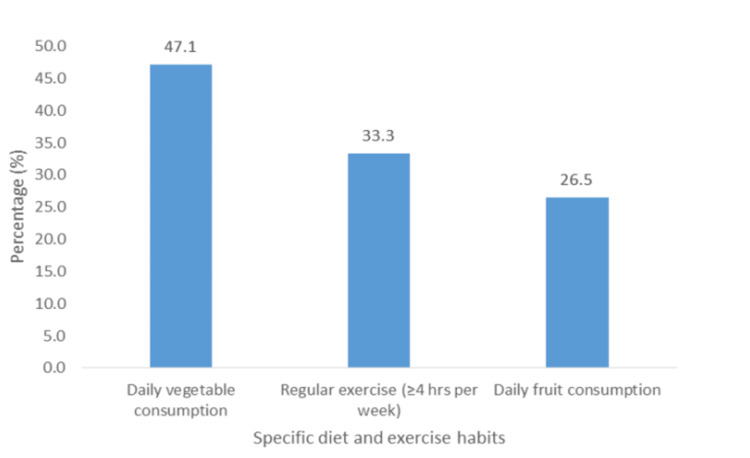
Specific diet and exercise habits of participants.

Almost half (47.1%; n = 48) of all participants consumed vegetables daily, and 26.5% (n = 27) consumed fruits daily (Figure 4). Only 6.9% (n = 7) used recreational drugs (cocaine, marijuana, lysergic acid diethylamide (LSD), etc.), 8.8% (n = 9) consumed alcohol, 16.7% (n = 17) smoked cigarettes, and 61.8% (n = 63) reported having a stressful life.

Comorbidities

Hypertension (30.4%; n = 31) and diabetes (18.6%; n = 19) were the leading comorbidities among participants. The prevalences by sex were 24.3% (n = 9/37) male and 33.8% (n = 22/65) female, and 13.5% (n = 5) male and 21.5% (n = 14) female, respectively, with no statistically significant differences (p = 0.375 and p = 0.430, respectively). Other medical conditions included COVID-19 (24.5%; n = 25), gastroesophageal reflux disease (5.9%; n = 6), anxiety (3.9%; n = 4), polycystic ovary syndrome (3.9%; n = 4), and depression (2.9%; n = 3). Other reported conditions with prevalences <2.0% (n = 1 or n = 2) included kidney stones, stroke, seizures, kidney failure, and scoliosis. No patient reported hypercholesterolemia or a sedentary lifestyle.

Psychosocial Factors

Instrument reliability/internal consistency: The reliability and internal consistency (Cronbach’s alpha) of each of the four instruments used to measure anxiety, depression, loneliness, and stress are shown in Table 2. 

**Table 2:** Instrument reliability and internal consistency. GAD-7: General Anxiety Disorder-7; UCLA-3: University of California, Los Angeles 3-Item Loneliness Scale; PHQ-9: Patient Health Questionnaire-9; PSS-10: Perceived Stress Scale.

Instrument (disorder)	Cronbach's alpha	Interpretation
GAD-7 (anxiety)	0.891	Good
PHQ-9 (depression)	0.850	Good
UCLA-3 (loneliness)	0.754	Acceptable
PSS-10 (stress)	0.819	Good
All items	0.914	Excellent

The reliability/internal consistency ranged from acceptable (0.754; UCLA_3) to good (0.891; GAD-7). The overall reliability was excellent (0.914; Table 2).

Table 3 shows Pearson’s product-moment bi-variable correlation coefficient among scores for the four attributes (anxiety, depression, loneliness, and stress). As shown, only one of the six bivariate correlations, namely that between loneliness (UCLA-3) and stress (PSS-10), was not statistically significant (r = 0.070, p = 0.473). Among the significant correlations, the strongest correlation occurred between anxiety (GAD-7) and depression (PHQ-9; r = 0.729, p ≤ 0.001); while the smallest was between loneliness and anxiety (r = 0.432; p ≤ 0.001).

**Table 3:** Correlation coefficients (with p-values).

	Correlation coefficient (p-value)				
Instrument (measure)	Anxiety		Depression	Loneliness	Stress
Anxiety	1	0.729 (≤0.001)		0.432 (≤0.001)	0.455 (≤0.001)
Depression			1	0.518 (≤0.001)	0.473 (≤0.001)
Loneliness				1	0.070 (0.483)
Stress					1

Summary Statistics: Attribute Scores

Table 4 shows selected summary statistics of anxiety, depression, loneliness, and stress scores. GAD-7, PHQ-9, and UCLA-3 scores were skewed left; while PSS-10 scores were normally distributed. 

**Table 4:** Selected summary statistics: attribute scores. GAD-7: General Anxiety Disorder-7; UCLA-3: University of California, Los Angeles 3-Item Loneliness Scale; PHQ-9: Patient Health Questionnaire-9; PSS-10: Perceived Stress Scale.

	Instrument/maximum possible score			
Statistic	GAD-7/21	PHQ-9/27	UCLA-3/9	PSS-10/40
n	102	102	102	102
Minimum	0.0	0.0	2.0	0.0
Median	5.0	6.0	4.0	19.0
Maximum	20.0	24.0	11.0	37.0
Mean	6.3	7.0	4.6	17.9
Standard deviation	5.50	5.50	1.89	7.16
Mode	0.0	2.0	1.79	19.0

Analysis of Variance (ANOVA): Total Attribute Scores

Table 5 shows the p-values obtained from the application of ANOVA methods to the total score for each of the four attributes. As seen, depression was the only attribute for which p ≤ 0.005. In other words, there were no statistically significant differences between, or among, mean depression scores for any of the sociodemographic variables of interest to the study. 

**Table 5:** P-values for analysis of variance (ANOVA).

	p-value			
Variable	Anxiety	Depression	Loneliness	Stress
Age group	0.130	0.383	0.906	0.244
Sex	0.685	0.825	0.599	0.711
Ethnicity	0.329	0.409	0.642	0.147
Education	0.160	0.632	0.494	0.064
Income	0.347	0.949	0.926	0.750
Employment status	0.166	0.967	0.719	0.257
Household composition	0.530	0.300	0.642	0.061
Financial support	0.116	0.405	0.296	0.404
Social support	0.615	0.700	0.595	0.029
Has comorbidities	0.045	0.079	0.017	0.534
Has diabetes	0.086	0.076	0.027	0.878
Hypertension	0.194	0.156	0.028	0.248

Multiple comparison methods (Tukey’s test) showed multiple associations, especially between anxiety and at least one comorbidity, social support, and less stress. For GAD-7 (anxiety), patients with at least one comorbidity had a significantly higher mean anxiety score than those without comorbidities (p = 0.045). For PHQ-9 (depression), there were no differences between or among the mean depression scores for any sociodemographic variables. For UCLA-3 (loneliness), the mean loneliness score of the patients with hypertension was significantly higher than that of the patients without hypertension (p = 0.028). Patients with no social support had a higher mean score than those with social support (p = 0.027); and patients with at least one comorbidity had a higher mean score than those with no comorbidities (p = 0.017). For PSS-10 (stress), the mean stress score of patients receiving social support was significantly lower than that of patients not receiving social support (p = 0.029).

Attribute Levels: Associated Factors and Predictors

**Figure 5:**
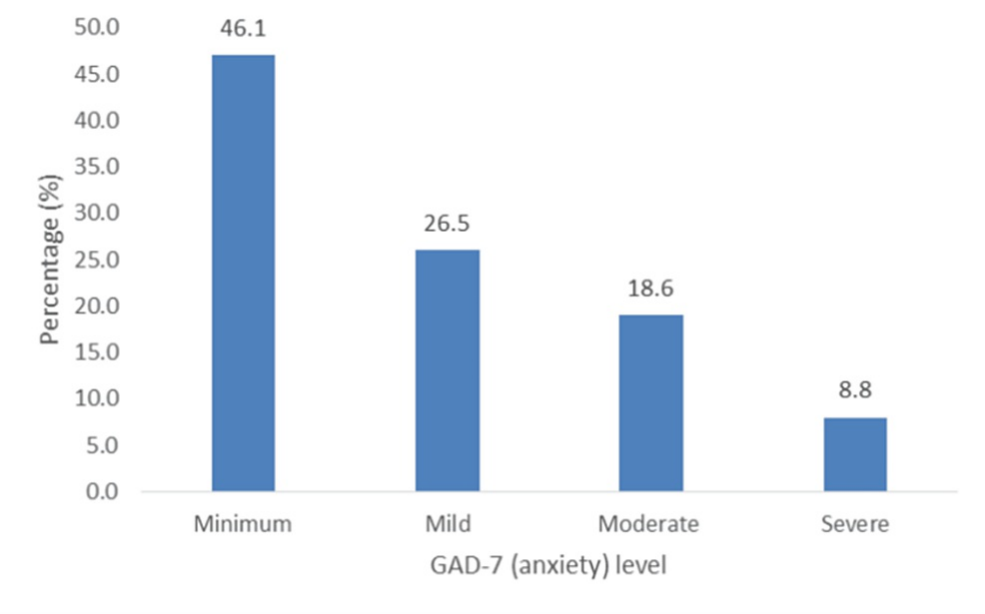
Percentage distribution of levels of anxiety (GAD-7). GAD-7: General Anxiety Disorder-7.

I. Anxiety (GAD-7): Patients’ anxiety levels ranged from mild to severe (53.9%; n = 55) (Figure 5). A stressful life was the only variable-associated factor (Chi-square: 10.754; df = 3; p = 0.013), and ordinal logistic regression showed that it was also a predictor of anxiety (adjusted odds ratio (AOR) = 4.222, p = 0.040; 95% CI (1.039, 5.030)). 

**Figure 6:**
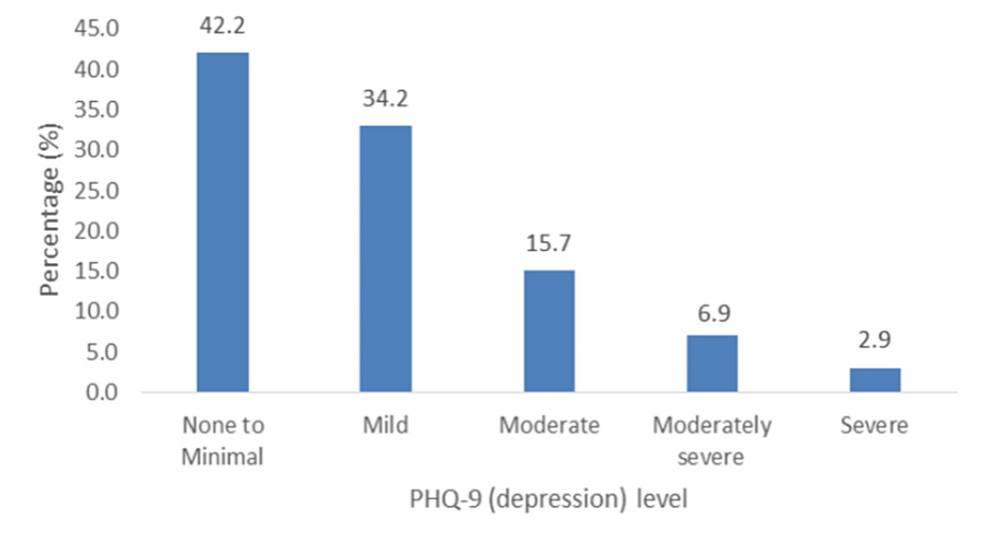
Percentage distribution of levels of depression (PHQ-9). PHQ-9: Patient Health Questionnaire-9.

II. Depression (PHQ-9): Figure 6 shows that levels of depression ranged from moderate to severe (25.5%; n = 26). There were no associated factors among the sociodemographic variables; hence, there were no predictors of depression.

III. Loneliness (UCLA-3): The prevalence of loneliness was 25.5% (n = 26), and stressful life was the only associated variable (Chi-square: 8.938, df = 1; p = 0.003). Binary logistic regression showed that it was also a predictor of loneliness (AOR = 6.081, p = 0.006; 95% CI (1.676, 22.072)).

**Figure 7:**
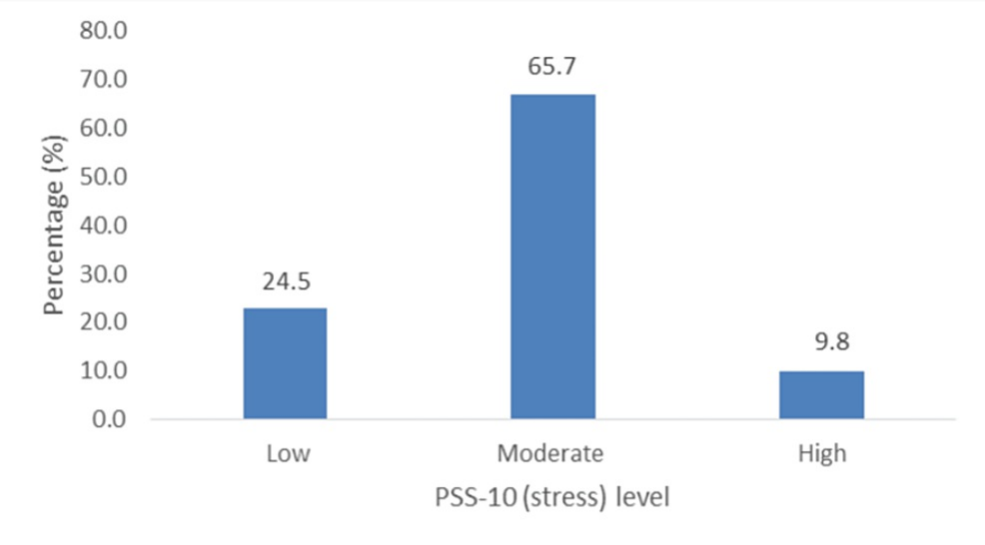
Percentage distribution of levels of stress (PSS-10). PSS-10: Perceived Stress Scale.

IV. Stress (PSS-10): Figure 7 shows that almost two-thirds of the patients (65.7%; n = 67) had moderate stress and that the prevalence of high stress was 9.8% (n = 10). However, the Chi-square analysis showed that there were no associated variables.

Consequences of patients with ACP

Psycho-Social-Financial

**Figure 8:**
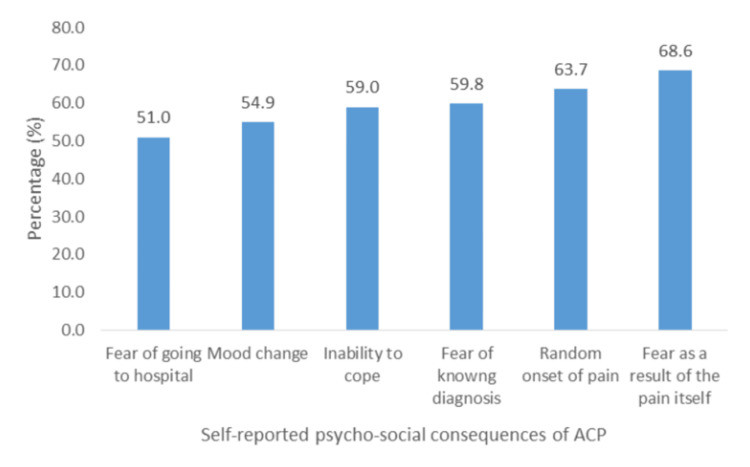
Self-reported psycho-social consequences of ACP. ACP: atypical chest pain.

Overall, "fear as a result of the pain" (68.6%; n = 69) was the most reported psycho-social consequence, and "fear of having to go to the hospital" (51.0%; n = 52) was the least reported (Figure 8).

Statistically significant associations were obtained between age group and "fear of knowing the diagnosis" (Chi-square: 8.364, df = 3, p = 0.04) and "changes in mood" (Chi-square: 8.095, df = 3, p = 0.044). Significant associations were also found between household composition and "fear of going to the hospital" (Chi-square: 11.508, df = 5, p = 0.042), "fear of knowing diagnosis" (Chi-square: 11.376, df = 5, p = 0.044), and "changes in mood" (Chi-square: 812.986, df = 5, p = 0.024). A significant association was additionally found between education level and "change in mood" (Chi-square: 7.408, df = 2, p = 0.025).

**Figure 9:**
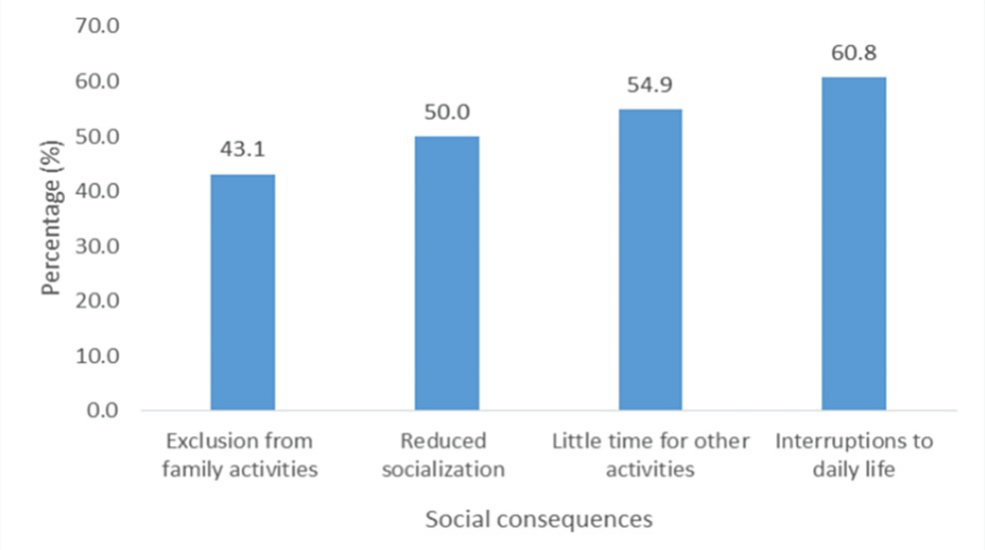
Social consequences of ACP. ACP: atypical chest pain.

The social consequence most reported was interruptions to daily life (60.8%; n = 61), followed by having little time for other activities (54.9%; n = 56), while the least reported was exclusion from family activities (43.1%; n = 44) (Figure 9).

Sex (Chi-square: 5.132, p = 0.013) and the highest level of education (Chi-square: 8.428, p = 0.015) were associated with reduced socialization, and monthly income was associated with reduced quality of life (Chi-square: 12.257, p = 0.031; Table 6).

**Table 6:** Predictors of reduced socialization. OR: odds ratio; CI: confidence interval.

			95% CI for OR	
Variable	OR	p	Lower	Upper
Sex				
Male	0.356	0.021	0.148	0.855
Female	1			
Education				
Primary	0.183	0.044	0.035	0.953
Secondary	1.748	0.223	0.72	4.102
Tertiary	1	0.379		

Reported recreational consequences were (i) reduction in time spent on hobbies (62.7%; n = 63), (ii) reduced daily physical activity (60.8%; n = 62), and (iii) reduction in time spent exercising (59.8%; n = 60). None of the study variables were associated with any of these consequences.

The reported financial consequences of ACP in order of incidence were job loss (31.4%; n = 32), financial costs as a result of switching healthcare providers (35.3%; n = 34), loss of income due to reduced attendance at work (47.1%; n = 48), costly treatment of modalities (56.9%; n = 58), and costly diagnostic/investigative tests (62.7%; n = 64).

Table 7 shows patients’ ethnicity, monthly income, whether or not they had social support, and the association of these factors with at least one of the financial consequences.

**Table 7:** Associations with financial consequences.

Variable	Associated variable (financial consequences)	Chi-square	df	p
Ethnicity	Switching health-care provider	11.474	3	0.008
	Costly investigative tests	7.849	3	0.049
	Costly treatment modalities	12.512	3	0.006
Monthly income	Cost of investigative tests	11.249	5	0.047
	Costly treatment of modalities	15.941	5	0.007
	Reduced attendance at work	18.952	5	0.002
Receive social support	Reduced attendance at work resulting in job loss	4.033	1	0.045

Predictors of Financial Consequences

Binary logistic regression showed that of the three demographic variables associated with financial consequences, monthly income was a predictor of both costly investigative tests and costly treatment modalities, and there were no predictors of reduced attendance at work (Table 8).

**Table 8:** Predictors of financial consequences. AOR: adjusted odds ratio; CI: confidence interval.

				95% CI for AOR	
Financial consequence	Monthly income ($TT)	AOR	p-value	Lower	Upper
Costly investigative tests	None	1			
	0-2500	1.488	0.594	0.345	6.418
	2500-5000	4.197	0.044	1.041	16.924
	5000-7500	4.169	0.071	0.884	19.654
	7500-10,000	0.795	0.721	0.225	2.803
	>10,000	1.536	0.636	0.260	9.080
Costly treatment modalities	None	1			
	0-2500	4.681	0.053	0.978	22.402
	2500-5000	12.342	0.001	2.772	54.957
	5000-7500	4.428	0.049	1.006	19.493
	7500-10,000	3.803	0.057	0.962	15.033
	>10,000	4.410	0.127	0.655	29.667

**Figure 10:**
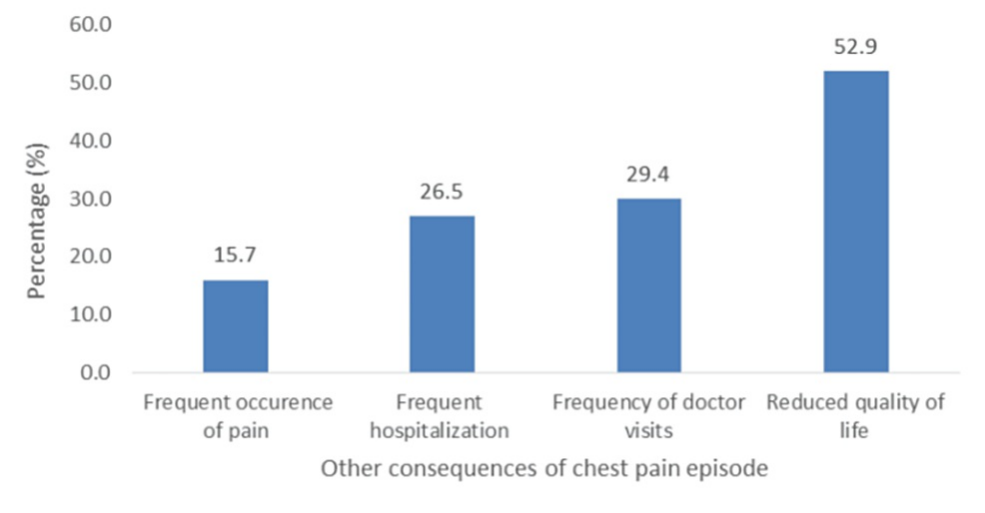
Reported consequences of chest pain episode.

The other reported consequences are shown in Figure 10. The least reported consequence was "frequent occurrence of pain" (5.7%; n =16), and the most reported was "reduced quality of life" (52.9%; n = 53). Monthly income was associated with reduced quality of life (Chi-square: 11.480, df = 5, p = 0.043); however, it was not a predictor.

Outcomes of patients with ACP

Treatment

**Figure 11:**
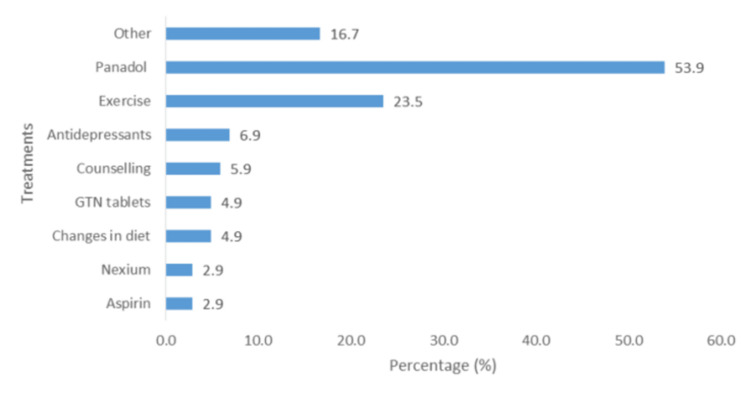
Treatments for ACP. ACP: atypical chest pain; GTN: glyceryl trinitrate.

The majority of patients used paracetamol (53.9%; n = 55), and 23.5% (n = 24) used exercise as a post-episode mechanism for self-management of their condition (Figure 11). Approximately 7% reported using antidepressants (6.9%; n = 7), and approximately 6% (5.9%; n = 6) sought some form of counseling. Other treatments mentioned, but not shown in the figure, due to small percentages of use (≤1.0; n = 1 or n = 2), included Daflon, Vastarel, enalapril, omeprazole, simvastatin, Isordil, Plavix, blood thinners, acid and gas tablets, and muscle relaxants.

## Discussion

Characteristics of patients with ACP

Socio-Demographics and Lifestyle

Patients were noted to be primarily women (63.7%) and of 31-50 years of age (43.1%), corroborating with Eslick et al.’s study [10], which found that 52.0% of patients with ACP were women. The study also found that the majority of patients with ACP had either secondary or tertiary school education (87.3%) and were employed full-time (56.9%) at the time of ACP onset. This study further showed a correlation between income and ACP, with the prevalence being highest in the salary bracket of TT $2500-$5000 (23.5%) and lowest in those earning a monthly income of more than TT $10000 (6.9%). This corroborates the findings of Wong et al. [11], who showed that prevalence declined with increasing income. 

The leading comorbidities were a medical history of hypertension (30.4%) or diabetes (18.6%). These findings contrast with the results of Lau et al. [12], who observed that there was an absence of diabetes, hypertension, or prior heart disease in 51.7% of CP patients. However, this correlates with the prevalence observed among patients with chest pain in the ED, as the majority had hypertension (44.5%) or diabetes (33.3%) [4].

The prevalence of lifestyle factors revealed some regular alcohol consumption (8.8%), smoking cigarettes (16.7%), and recreational drugs (6.9%). Smoking prevalence was low in this study compared with a study by Wilhelmsen et al. [13], who found that 41% of participants who engaged in smoking also presented with ACP. Furthermore, in a study by Karlaftis et al. [14], the results showed that 23% of patients with ACP consumed alcohol. However, our study revealed that only 8.8% of the study population engaged in alcohol consumption. Additionally, 66.7%, 73.5%, and 52.9% of the sample did not engage in regular exercise, consume fruits daily, and consume vegetables daily, respectively. 

Psychosocial Factors Prevalence and Associations

Our study revealed that the majority of participants experienced at least mild anxiety, and a significant number experienced moderate depression, loneliness, and stress. McDevitt-Petrovic et al. further asserted a correlation between psychological difficulties such as anxiety, depression, panic disorders, and chest pain [15]. Most patients had mild to severe anxiety (53.9%), and moderate to severe anxiety occurred in 27.4% of participants. This contrasts with a study by Wertli et al. [16], which highlighted that anxiety disorders are rarely considered a possible cause of ACP by physicians. However, this is notably different from a study by Demiryoguran et al. [17], which stated that 70% of patients had a high Hospital Anxiety and Depression Scale or anxiety score. In this study, a strong association was observed between anxiety and perceived stress. Our results indicated a prevalence of moderate to severe depression of 25.5% was lower than that reported by Fagring et al. [18] and Lin et al. [19], who found that depression was self-reported in 35% and 50% of the men and women, and in 61.5% of the sample, respectively. This contrasts with the much lower depression levels reported by Eslick et al. [10], who documented the prevalence of clinical depression as 7%. The prevalence of loneliness in this study was 25.5%. Previous studies have demonstrated a correlation between loneliness and ACP [20]. Paul et al. [21] posited that loneliness is more common in women and those with lower socioeconomic status. This study showed that 65.7% of the patients with ACP had moderate stress, and 9.8% had high stress. A previous study by Bahall et al. [4] among patients with chest pain at the ED showed that only 2% self-reported having a stressful life. In the present study, stress was significantly associated with anxiety and loneliness. Kim et al. reported a positive correlation between stress [22] in patients with recurrent chest pain.

Consequences of patients with ACP

Psycho-Social-Financial

Overall, participants were psychologically, socially, and financially affected by ACP. In our study, there was a general fear of pain, knowing or not knowing the diagnosis, and visiting the hospital. Participants’ greatest fear was "as a result of the pain" (68.6%), and the least but still considerably high was "fear of having to go to the hospital" (51.0%). Similar fear has been reported in other studies [23]. The most commonly reported social consequences of ACP were interruptions to daily life (60.8%), followed by having little time for other activities (54.9%), reduced socialization (50.0%), and exclusion from family activities (43.1%). This correlates well with a study by Eslick and Talley, who reported interruptions in daily activities in 63% of participants [24]. In this study, there was also a significant reduction in time spent on hobbies (62.7%). Due to uncertainty and attempts to clarify their diagnosis, patients reported costly treatment (56.9%) and costly diagnostic/investigative tests (62.7%) as the most common financial consequences. This finding was similar to that of Eslick et al., who reported a high cost of treatment [25]. We found that monthly income was a predictor of costly investigative tests and treatment modalities. The majority of participants reported "reduced quality of life" (52.9%), similar to the low quality of life reported by patients with ACP found by Mourad et al. [26]. Another study by Eslick et al. found that 36% of patients with ACP reported much lower quality of life levels [25].

Outcomes of patients with ACP

Treatment

The majority of patients used paracetamol (53.9%), and 23.5% used exercise as a post-episode mechanism for self-management of their condition. Other studies have reported that commonly used drugs include proton pump inhibitors, muscle relaxants (nitrates, calcium channel blockers), and pain modulators [27].

Limitations

This study had multiple limitations. This included inappropriate coding of hospital files leading to the rejection of many patients and communication difficulties because of outdated patient phone numbers in the files. Much of the data were based on recall and subjective responses, which carry a certain amount of bias. One significant disadvantage of the study is that it did not include certain anthropometric chest measurements, which have a substantial correlation with ACP [28].

Recommendations

Patients presenting with non-cardiac chest pain should be evaluated using a test tool to diagnose the risk. There is a need for a comprehensive plan across the country to manage psychosocial issues, particularly for high-risk populations.

## Conclusions

Atypical chest pain was common in middle-aged East Indian females who completed secondary education and had poor socioeconomic status. A minority of patients were diabetic and hypertensive and had a poor lifestyle. Most participants had psychological difficulties, including anxiety. Although ACP may be benign, most patients experienced curtailment of social and leisure activities, and many suffered financial setbacks. Most patients were treated with analgesics and lifestyle recommendations, such as exercise. Future studies are needed to further explore the profiles of patients with ACP. Additional research on patients with ACP utilizing diagnostic scales is required to determine whether there is a relationship between ACP and other factors such as dietary practices, hormonal imbalances, traumatic experiences, eating disorders, and genetic factors.
